# 2-[Hy­droxy(4-meth­oxy­phen­yl)methyl­idene]indane-1,3-dione

**DOI:** 10.1107/S1600536812021824

**Published:** 2012-05-26

**Authors:** Jing-Chi Gu, Bi-Xue Zhu, Yun-Qian Zhang

**Affiliations:** a Key Laboratory of Macrocyclic and Supramolecular Chemistry of Guizhou Province, Guizhou University, Guiyang 550025, People’s Republic of China.

## Abstract

In the title compound, C_17_H_12_O_4_, there is an intra­molecular O—H⋯O hydrogen bond. The dihedral angle between the indane ring system [maximun deviation = 0.023 (2) Å] and the benzene ring is 37.42 (9)°.

## Related literature

For general background to the synthesis and pharmacological properties of 1,3-indandione derivatives, see: Cheng *et al.* (2011 ▶).
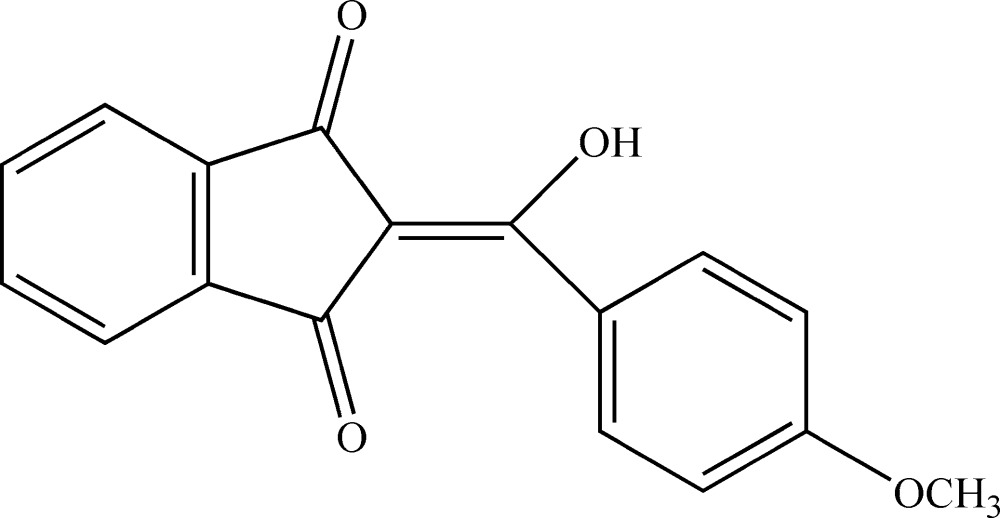


## Experimental

### 

#### Crystal data

C_17_H_12_O_4_
*M* *_r_* = 280.27
Monoclinic, 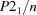

*a* = 17.779 (4) Å
*b* = 3.8405 (9) Å
*c* = 19.026 (4) Å
β = 92.984 (8)°
*V* = 1297.4 (5) Å^3^
*Z* = 4
Mo *K*α radiation
μ = 0.10 mm^−1^
*T* = 293 K
0.23 × 0.19 × 0.18 mm

#### Data collection

Bruker SMART CCD area-detector diffractometer
Absorption correction: multi-scan (*SADABS*; Bruker, 2005 ▶) *T*_min_ = 0.977, *T*_max_ = 0.982
10521 measured reflections
2311 independent reflections
1553 reflections with *I* > 2σ(*I*)
*R*_int_ = 0.077

#### Refinement

*R*[*F* ^2^ > 2σ(*F* ^2^)] = 0.049
*wR*(*F* ^2^) = 0.127
*S* = 0.97
2311 reflections
191 parameters
H-atom parameters constrained
Δρ_max_ = 0.21 e Å^−3^
Δρ_min_ = −0.17 e Å^−3^

### 

Data collection: *SMART* (Bruker, 2002 ▶); cell refinement: *SAINT* (Bruker, 2002 ▶); data reduction: *SAINT*; program(s) used to solve structure: *SHELXS97* (Sheldrick, 2008 ▶); program(s) used to refine structure: *SHELXL97* (Sheldrick, 2008 ▶); molecular graphics: *ORTEP-3 for Windows* (Farrugia, 1997 ▶); software used to prepare material for publication: *WinGX* (Farrugia, 1999 ▶).

## Supplementary Material

Crystal structure: contains datablock(s) global, I. DOI: 10.1107/S1600536812021824/nc2275sup1.cif

Structure factors: contains datablock(s) I. DOI: 10.1107/S1600536812021824/nc2275Isup2.hkl

Supplementary material file. DOI: 10.1107/S1600536812021824/nc2275Isup3.cml

Additional supplementary materials:  crystallographic information; 3D view; checkCIF report

## Figures and Tables

**Table 1:** Hydrogen-bond geometry (Å, °)

*D*—H⋯*A*	*D*—H	H⋯*A*	*D*⋯*A*	*D*—H⋯*A*
O1—H1⋯O2	0.82	1.78	2.539 (2)	152

**Fig. 1.**
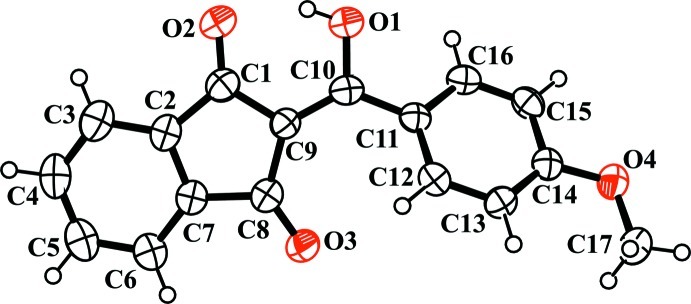
The molecular structure of the title compound showing the atom-labelling scheme. Displacement ellipsoids are drawn at the 50% probability level.

## supplementary crystallographic information

### Comment

In general, 1,3-indandione derivatives demonstrate an anticoagulant properties. The synthesis and pharmacological properties of some chemicals of this category have been reported (Dolmella *et al.*, 1961). The prepare of derivatives containing 2*H*-indene-1,3-dione unit have received very substantial attention (Cheng *et al.*, 2011). In view of this biological importance a part of our ongoing studies of 1,3-indandione derivatives includes the crystal structure determination of the title compound. The molecule of the title compound shows non-coplanar structure (Fig. 1). An intramolecular O—H···O hydrogen bonds is observed (Table 1), which links the hydroxyl oxygen to the nearby keto-oxygen atom of the 2*H*-indene-1,3-dione unit, forming a planar six-membered ring. The dihedral angle between the six-membered ring and the plane of 2*H*-indene-1,3-dione unit is 173.94°, and the dihedral angle between the six-membered ring and the benzene ring is 148.51°.

### Experimental

*p*-methoxy-acetophenone (166 mg, 1.2 mmol) in tetrahydrofuran (15 ml) was added slowly with stirring to dimethyl phthalate(232.8 mg, 1.2 mmol) and NaH (120 mg, 5 mmol) in THF (30 ml) and the mixture was heated at reflux for 12 h. The solution was allowed to cool and the THF was removed partly under reduced pressure. The precipitate was collected by filtration and washed with waterand dried; the residue was crystallized from CHCl_3_ to afford the title compound as a yellow solid [yield 65%, m.p. 398–400 K]. Single crystal suitable for X-ray diffraction was prepared by slow evaporation of a solution of the title compound in methanol at room temperature.

### Refinement

All H atoms were geometrically positioned(C—H = 0.93–0.96 Å) and treated as riding, with *U*_iso_(H) = 1.2–1.5 *U*_eq_.

### Crystal data

**Table:**

C_17_H_12_O_4_	*F*(000) = 584
*M**_r_* = 280.27	*D*_x_ = 1.435 Mg m^−^^3^
Monoclinic, *P*2_1_/*n*	Mo *K*α radiation, λ = 0.71073 Å
Hall symbol: -P 2yn	Cell parameters from 2311 reflections
*a* = 17.779 (4) Å	θ = 1.5–25.1°
*b* = 3.8405 (9) Å	µ = 0.10 mm^−^^1^
*c* = 19.026 (4) Å	*T* = 293 K
β = 92.984 (8)°	Block, colorless
*V* = 1297.4 (5) Å^3^	0.23 × 0.19 × 0.18 mm
*Z* = 4	

### Data collection

**Table:**

Bruker SMART CCD area-detector diffractometer	2311 independent reflections
Radiation source: fine-focus sealed tube	1553 reflections with *I* > 2σ(*I*)
Graphite monochromator	*R*_int_ = 0.077
φ and ω scan	θ_max_ = 25.1°, θ_min_ = 1.5°
Absorption correction: multi-scan (*SADABS*; Bruker, 2005)	*h* = −21→19
*T*_min_ = 0.977, *T*_max_ = 0.982	*k* = −4→4
10521 measured reflections	*l* = −22→22

### Refinement

**Table:**

Refinement on *F*^2^	Primary atom site location: structure-invariant direct methods
Least-squares matrix: full	Secondary atom site location: difference Fourier map
*R*[*F*^2^ > 2σ(*F*^2^)] = 0.049	Hydrogen site location: inferred from neighbouring sites
*wR*(*F*^2^) = 0.127	H-atom parameters constrained
*S* = 0.97	*w* = 1/[σ^2^(*F*_o_^2^) + (0.0678*P*)^2^] where *P* = (*F*_o_^2^ + 2*F*_c_^2^)/3
2311 reflections	(Δ/σ)_max_ < 0.001
191 parameters	Δρ_max_ = 0.21 e Å^−^^3^
0 restraints	Δρ_min_ = −0.17 e Å^−^^3^

### Special details

**Table:**

Geometry. All esds (except the esd in the dihedral angle between two l.s. planes) are estimated using the full covariance matrix. The cell esds are taken into account individually in the estimation of esds in distances, angles and torsion angles; correlations between esds in cell parameters are only used when they are defined by crystal symmetry. An approximate (isotropic) treatment of cell esds is used for estimating esds involving l.s. planes.
Refinement. Refinement of F^2^ against ALL reflections. The weighted R-factor wR and goodness of fit S are based on F^2^, conventional R-factors R are based on F, with F set to zero for negative F^2^. The threshold expression of F^2^ > 2sigma(F^2^) is used only for calculating R-factors(gt) etc. and is not relevant to the choice of reflections for refinement. R-factors based on F^2^ are statistically about twice as large as those based on F, and R- factors based on ALL data will be even larger.

### Fractional atomic coordinates and isotropic or equivalent isotropic displacement parameters (Å^2^)

**Table:**

	*x*	*y*	*z*	*U*_iso_*/*U*_eq_	
C1	0.51739 (13)	0.1113 (6)	0.36369 (11)	0.0431 (6)	
C2	0.44114 (13)	0.2488 (6)	0.37373 (11)	0.0407 (6)	
C3	0.40844 (16)	0.3680 (6)	0.43398 (12)	0.0551 (7)	
H3	0.4347	0.3647	0.4775	0.066*	
C4	0.33527 (16)	0.4919 (7)	0.42678 (14)	0.0561 (7)	
H4	0.3119	0.5734	0.4662	0.067*	
C5	0.29666 (15)	0.4961 (6)	0.36209 (13)	0.0513 (7)	
H5	0.2477	0.5817	0.3587	0.062*	
C6	0.32898 (13)	0.3762 (6)	0.30215 (12)	0.0448 (6)	
H6	0.3026	0.3810	0.2586	0.054*	
C7	0.40147 (12)	0.2492 (5)	0.30887 (10)	0.0370 (5)	
C8	0.44965 (13)	0.1018 (5)	0.25401 (11)	0.0366 (5)	
C9	0.52302 (12)	0.0199 (5)	0.28999 (10)	0.0353 (5)	
C10	0.59134 (12)	−0.0856 (5)	0.26557 (11)	0.0374 (6)	
C11	0.61151 (12)	−0.1687 (5)	0.19378 (10)	0.0338 (5)	
C12	0.56158 (12)	−0.3235 (5)	0.14478 (11)	0.0375 (5)	
H12	0.5125	−0.3693	0.1568	0.045*	
C13	0.58354 (13)	−0.4110 (5)	0.07820 (11)	0.0378 (6)	
H13	0.5495	−0.5174	0.0462	0.045*	
C14	0.65592 (12)	−0.3404 (5)	0.05943 (10)	0.0363 (5)	
C15	0.70668 (12)	−0.1831 (6)	0.10769 (11)	0.0401 (6)	
H15	0.7552	−0.1315	0.0949	0.048*	
C16	0.68528 (12)	−0.1036 (5)	0.17427 (11)	0.0391 (6)	
H16	0.7201	−0.0057	0.2067	0.047*	
C17	0.63265 (14)	−0.5648 (7)	−0.05674 (12)	0.0503 (7)	
H17A	0.6587	−0.6058	−0.0989	0.075*	
H17B	0.5915	−0.4073	−0.0667	0.075*	
H17C	0.6136	−0.7813	−0.0398	0.075*	
O1	0.65026 (9)	−0.1081 (5)	0.31159 (8)	0.0537 (5)	
H1	0.6368	−0.0636	0.3512	0.081*	
O2	0.56922 (10)	0.0915 (5)	0.40994 (8)	0.0609 (5)	
O3	0.42812 (9)	0.0644 (4)	0.19222 (8)	0.0477 (5)	
O4	0.68340 (9)	−0.4164 (4)	−0.00444 (7)	0.0470 (4)	

### Atomic displacement parameters (Å^2^)

**Table:**

	*U*^11^	*U*^22^	*U*^33^	*U*^12^	*U*^13^	*U*^23^
C1	0.0489 (15)	0.0397 (14)	0.0407 (13)	−0.0010 (11)	0.0010 (12)	0.0020 (10)
C2	0.0521 (15)	0.0316 (13)	0.0389 (12)	−0.0033 (11)	0.0086 (11)	0.0000 (10)
C3	0.0693 (19)	0.0524 (16)	0.0443 (14)	−0.0007 (14)	0.0100 (13)	0.0003 (12)
C4	0.0665 (19)	0.0485 (16)	0.0553 (16)	0.0035 (14)	0.0233 (14)	−0.0022 (12)
C5	0.0532 (16)	0.0361 (14)	0.0661 (18)	0.0039 (12)	0.0179 (14)	0.0051 (12)
C6	0.0484 (15)	0.0346 (14)	0.0520 (14)	0.0016 (11)	0.0074 (12)	0.0033 (10)
C7	0.0459 (15)	0.0246 (12)	0.0407 (12)	−0.0029 (10)	0.0057 (11)	0.0026 (9)
C8	0.0479 (14)	0.0245 (12)	0.0376 (13)	−0.0026 (10)	0.0037 (11)	0.0050 (9)
C9	0.0403 (14)	0.0312 (12)	0.0344 (12)	0.0012 (10)	0.0010 (10)	−0.0003 (9)
C10	0.0414 (14)	0.0291 (12)	0.0410 (12)	−0.0007 (10)	−0.0053 (11)	0.0015 (9)
C11	0.0369 (13)	0.0255 (11)	0.0389 (12)	0.0034 (10)	−0.0010 (10)	0.0034 (9)
C12	0.0366 (13)	0.0303 (12)	0.0459 (13)	−0.0029 (10)	0.0048 (11)	0.0022 (10)
C13	0.0431 (14)	0.0307 (12)	0.0393 (12)	−0.0037 (10)	0.0000 (11)	−0.0017 (9)
C14	0.0418 (14)	0.0281 (12)	0.0390 (12)	0.0042 (10)	0.0035 (11)	0.0043 (9)
C15	0.0336 (13)	0.0383 (13)	0.0488 (13)	−0.0013 (10)	0.0052 (11)	0.0060 (10)
C16	0.0379 (13)	0.0350 (13)	0.0438 (13)	−0.0011 (10)	−0.0045 (11)	0.0022 (10)
C17	0.0592 (16)	0.0494 (16)	0.0423 (13)	−0.0001 (12)	0.0042 (12)	−0.0057 (11)
O1	0.0471 (10)	0.0711 (13)	0.0420 (9)	0.0095 (9)	−0.0056 (8)	−0.0058 (8)
O2	0.0582 (12)	0.0835 (14)	0.0400 (9)	0.0096 (10)	−0.0059 (9)	−0.0056 (8)
O3	0.0501 (10)	0.0543 (11)	0.0384 (9)	0.0066 (8)	−0.0017 (8)	−0.0009 (7)
O4	0.0474 (10)	0.0535 (11)	0.0406 (9)	0.0002 (8)	0.0073 (8)	−0.0030 (7)

### Geometric parameters (Å, º)

**Table:**

C1—O2	1.243 (3)	C10—C11	1.465 (3)
C1—C9	1.454 (3)	C11—C12	1.387 (3)
C1—C2	1.476 (3)	C11—C16	1.404 (3)
C2—C7	1.389 (3)	C12—C13	1.386 (3)
C2—C3	1.390 (3)	C12—H12	0.9300
C3—C4	1.385 (4)	C13—C14	1.380 (3)
C3—H3	0.9300	C13—H13	0.9300
C4—C5	1.378 (3)	C14—O4	1.365 (2)
C4—H4	0.9300	C14—C15	1.392 (3)
C5—C6	1.382 (3)	C15—C16	1.376 (3)
C5—H5	0.9300	C15—H15	0.9300
C6—C7	1.378 (3)	C16—H16	0.9300
C6—H6	0.9300	C17—O4	1.427 (3)
C7—C8	1.496 (3)	C17—H17A	0.9600
C8—O3	1.226 (2)	C17—H17B	0.9600
C8—C9	1.475 (3)	C17—H17C	0.9600
C9—C10	1.384 (3)	O1—H1	0.8200
C10—O1	1.333 (2)		
			
O2—C1—C9	125.6 (2)	O1—C10—C11	112.11 (19)
O2—C1—C2	125.6 (2)	C9—C10—C11	129.68 (19)
C9—C1—C2	108.71 (19)	C12—C11—C16	118.2 (2)
C7—C2—C3	121.2 (2)	C12—C11—C10	122.7 (2)
C7—C2—C1	108.22 (19)	C16—C11—C10	119.01 (19)
C3—C2—C1	130.6 (2)	C13—C12—C11	121.1 (2)
C4—C3—C2	117.6 (2)	C13—C12—H12	119.4
C4—C3—H3	121.2	C11—C12—H12	119.4
C2—C3—H3	121.2	C14—C13—C12	120.0 (2)
C5—C4—C3	120.9 (2)	C14—C13—H13	120.0
C5—C4—H4	119.5	C12—C13—H13	120.0
C3—C4—H4	119.5	O4—C14—C13	124.76 (19)
C4—C5—C6	121.6 (2)	O4—C14—C15	115.50 (19)
C4—C5—H5	119.2	C13—C14—C15	119.7 (2)
C6—C5—H5	119.2	C16—C15—C14	120.2 (2)
C7—C6—C5	117.9 (2)	C16—C15—H15	119.9
C7—C6—H6	121.0	C14—C15—H15	119.9
C5—C6—H6	121.0	C15—C16—C11	120.7 (2)
C6—C7—C2	120.8 (2)	C15—C16—H16	119.6
C6—C7—C8	129.5 (2)	C11—C16—H16	119.6
C2—C7—C8	109.7 (2)	O4—C17—H17A	109.5
O3—C8—C9	130.1 (2)	O4—C17—H17B	109.5
O3—C8—C7	123.5 (2)	H17A—C17—H17B	109.5
C9—C8—C7	106.35 (17)	O4—C17—H17C	109.5
C10—C9—C1	120.02 (19)	H17A—C17—H17C	109.5
C10—C9—C8	132.62 (19)	H17B—C17—H17C	109.5
C1—C9—C8	107.00 (19)	C10—O1—H1	109.5
O1—C10—C9	118.17 (18)	C14—O4—C17	117.63 (17)
			
O2—C1—C2—C7	176.0 (2)	C7—C8—C9—C10	−172.1 (2)
C9—C1—C2—C7	−1.1 (2)	O3—C8—C9—C1	−179.1 (2)
O2—C1—C2—C3	−3.3 (4)	C7—C8—C9—C1	0.9 (2)
C9—C1—C2—C3	179.6 (2)	C1—C9—C10—O1	1.6 (3)
C7—C2—C3—C4	−0.9 (4)	C8—C9—C10—O1	173.8 (2)
C1—C2—C3—C4	178.3 (2)	C1—C9—C10—C11	−175.8 (2)
C2—C3—C4—C5	0.0 (4)	C8—C9—C10—C11	−3.6 (4)
C3—C4—C5—C6	0.3 (4)	O1—C10—C11—C12	148.4 (2)
C4—C5—C6—C7	0.3 (3)	C9—C10—C11—C12	−34.1 (3)
C5—C6—C7—C2	−1.3 (3)	O1—C10—C11—C16	−28.8 (3)
C5—C6—C7—C8	179.4 (2)	C9—C10—C11—C16	148.8 (2)
C3—C2—C7—C6	1.6 (3)	C16—C11—C12—C13	−0.2 (3)
C1—C2—C7—C6	−177.80 (19)	C10—C11—C12—C13	−177.40 (18)
C3—C2—C7—C8	−179.0 (2)	C11—C12—C13—C14	−0.8 (3)
C1—C2—C7—C8	1.6 (2)	C12—C13—C14—O4	179.78 (18)
C6—C7—C8—O3	−2.3 (4)	C12—C13—C14—C15	0.4 (3)
C2—C7—C8—O3	178.4 (2)	O4—C14—C15—C16	−178.35 (18)
C6—C7—C8—C9	177.8 (2)	C13—C14—C15—C16	1.1 (3)
C2—C7—C8—C9	−1.6 (2)	C14—C15—C16—C11	−2.2 (3)
O2—C1—C9—C10	−3.0 (3)	C12—C11—C16—C15	1.7 (3)
C2—C1—C9—C10	174.07 (19)	C10—C11—C16—C15	179.00 (18)
O2—C1—C9—C8	−177.0 (2)	C13—C14—O4—C17	3.1 (3)
C2—C1—C9—C8	0.1 (2)	C15—C14—O4—C17	−177.49 (19)
O3—C8—C9—C10	8.0 (4)		

### Hydrogen-bond geometry (Å, º)

**Table:**

*D*—H···*A*	*D*—H	H···*A*	*D*···*A*	*D*—H···*A*
O1—H1···O2	0.82	1.78	2.539 (2)	152
